# Doxorubicin-Induced Modulation of TGF-β Signaling Cascade in Mouse Fibroblasts: Insights into Cardiotoxicity Mechanisms

**DOI:** 10.21203/rs.3.rs-3186393/v1

**Published:** 2023-07-27

**Authors:** Conner Patricelli, Parker Lehmann, Julia Thom Oxford, Xinzhu Pu

**Affiliations:** Boise State University; Idaho College of Osteopathic Medicine; Boise State University; Boise State University

## Abstract

Doxorubicin (DOX)-induced cardiotoxicity has been widely observed, yet the specific impact on cardiac fibroblasts is not fully understood. Additionally, the modulation of the transforming growth factor beta (TGF-β) signaling pathway by DOX remains to be fully elucidated. This study investigated DOX’s ability to modulate the expression of genes and proteins involved in the TGF-β signaling cascade in mouse fibroblasts from two sources by assessing the impact of DOX treatment on TGF-β inducible expression of pivotal genes and proteins within fibroblasts. Mouse embryonic fibroblasts (NIH3T3) and mouse primary cardiac fibroblasts (CFs) were treated with DOX in the presence of TGF-β1 to assess changes in protein levels by western blot and changes in mRNA levels by quantitative reverse transcriptase polymerase chain reaction (qRT-PCR). Our results revealed a dose-dependent reduction in cellular communication network factor 2 (CCN2) protein levels upon DOX treatment in both NIH3T3 and CFs. Moreover, we observed that DOX inhibited the TGF-β1 induced expression of BMP1 in NIH3T3 cells, while BMP1 levels remained high in CFs, and that TGF-β1 induces the phosphorylation of SMAD2 in both NIH3T3 cells and CFs. While DOX treatment diminished the extent of phosphorylation, the reduction did not reach statistical significance. DOX also inhibited the TGF-β1 induced expression of COL1 in NIH3T3 cells and CFs. Finally, DOX inhibited the TGF-β1 induced expression of Atf4 and increased the expression of Cdkn1a, Id1, Id2, Runx1, Tgfb1, Inhba, Thbs1, Bmp1, and Stat1 in NIH3T3 cells but not CFs, indicating the potential for cell-specific responses to DOX and its modulation of the TGF-β signaling pathway. Understanding the underlying mechanisms of the ability of DOX to modulate gene expression and signaling pathways in fibroblasts holds promise for future development of targeted therapeutic strategies to mitigate DOX-induced cardiotoxicity specifically affecting CFs.

## Introduction

Chemotherapy has improved cancer survival rates, however, side effects to chemotherapeutic drugs remains a concern. An increased risk of cardiovascular complications and cardiotoxicity is attributed to anthracyclines ^1–4^. Anthracyclines are a class of naturally occurring antibiotics produced by Streptomyces bacteria that exhibit broad anticancer efficacy. One of the most widely used anthracyclines is DOX, which was approved by the FDA in 1974 for the treatment of cancers, including hematopoietic cancers such as leukemias and lymphomas, and metastatic solid tumors such as breast, gastric, neuroblastoma, lung, ovarian, and bladder cancers in both adult and pediatric patients ^5^. Despite more than five decades of research, the clinical use of DOX is limited by its dose-dependent cardiotoxic side effects, which remain a serious risk to patients.

Echocardiography and cardiac magnetic resonance imaging are standard methods used to monitor the left ventricle ejection fraction (LVEF) to assess the progression of cardiotoxicity induced by DOX ^6–10^. DOX-induced cardiotoxicity is characterized by a gradual decline in LVEF which, if not managed, can lead to heart failure. A significant reduction in LVEF is defined as a decrease of more than 10% from baseline to a value below the lower limit of normal (usually less than 50–55%) ^10–12^. The risk of developing cardiotoxicity increases with the cumulative dose of DOX ^13^. The reported risk of DOX-induced cardiotoxicity by cumulative dose is 3–5% for 400 mg/m^2^, 7–26% for 550 mg/m^2^, and 18–48% for 700 mg/m^2 14–18^. Moreover, age is a significant factor that contributes to the susceptibility to cardiotoxicity in DOX-treated patients. Patients under five years old or over sixty-five years old are more vulnerable to developing cardiotoxicity ^14,19^. It is likely that these age groups are more prone to cardiotoxicity due to age-related differences in the cardiovascular system which also increase the risk of developing heart failure.

DOX exerts its antitumor activity by intercalating into DNA, which inhibits the binding of topoisomerase IIβ and the unwinding of supercoiled DNA ^20–23^. This inhibition leads to a blockage of DNA synthesis and the eventual induction of double-stranded DNA breaks, triggering programmed cell death in cells ^24,25^. Despite its therapeutic benefits, DOX is associated with off-target effects, which include the production of reactive oxygen species (ROS). The precise mechanisms underlying DOX-induced ROS overproduction are not fully understood, however, studies suggest that mitochondrial dysfunction ^26,27^, calcium homeostasis ^28,29^, iron chelation ^30,31^, and inflammatory responses ^32–35^ are implicated in ROS generation caused by DOX. Although antioxidants have been used in combination with DOX to decrease the buildup of free radicals, this approach has not proven effective in reducing cardiotoxicity while maintaining the antitumor efficacy of DOX ^36^. As a result, the need for alternative therapeutic options to lower the risk of cardiotoxicity from DOX remains.

DOX-induced cardiotoxicity research has primarily focused on cardiomyocytes. The impact of DOX on CFs remains underexplored. The myocardium is a complex tissue comprising multiple cell types, including CFs, which are the most abundant cell type in the left ventricle of the heart ^37^. CFs play a crucial role in regulating the expression and accumulation of extracellular matrix (ECM) components such as collagens, fibronectin, matrix metalloproteinases (MMPs), and endogenous protease inhibitors known as tissue inhibitors of metalloproteinases (TIMPs) ^38–42^. Despite their importance, the study of CFs in DOX-induced cardiotoxicity has been limited compared to cardiomyocytes. Therefore, there is a need to assess the contribution of CFs to DOX-induced cardiotoxicity and bridge the knowledge gap in this area. Transforming growth factor β (TGF-β) is a pivotal cytokine/growth factor that has been shown to stimulate fibroblasts ^43,44^. The TGF-β signaling pathway has been implicated in fibrosis in the myocardium, which contributes to cardiac dysfunction ^45–49^. In this study, we investigated the effects of DOX on TGF-β signaling cascade in mouse fibroblasts from two sources to better understand the mechanisms of its cardiotoxicity, which in turn may facilitate the identification of potential therapeutic targets to alleviate this serious adverse effect of DOX.

## Results

### Effect of DOX on fibroblast viability

**Figure 1:**
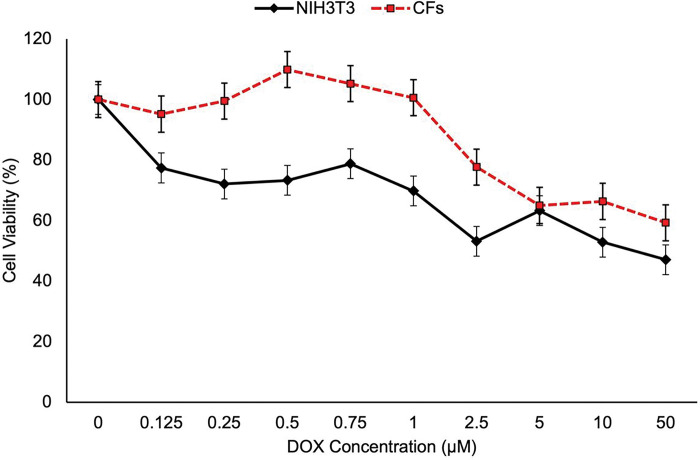
Cell viability remains high at DOX concentrations less than 1 μM. Cell viability analysis was performed using the alamarBlue assay on NIH3T3 cells and CFs exposed to specific concentrations of DOX for 24 hours. Error bars represent ± standard error represented a sample size of n=8 for NIH3T3 and n=6 for CFs.

Cell viability of NIH3T3 and CFs was evaluated using an alamarBlue assay. Cells were treated with 0–50 μM DOX for 24 hours. A significant decrease in cell viability was observed at 2.5 μM DOX and above (Fig. 1). Thus, sublethal concentrations of DOX (≤ 1.0 μM) were used in the subsequent experiments.

### DOX treatment Inhibited TGF-β induction of CCN2 protein level in a dose-dependent manner

**Figure 2:**
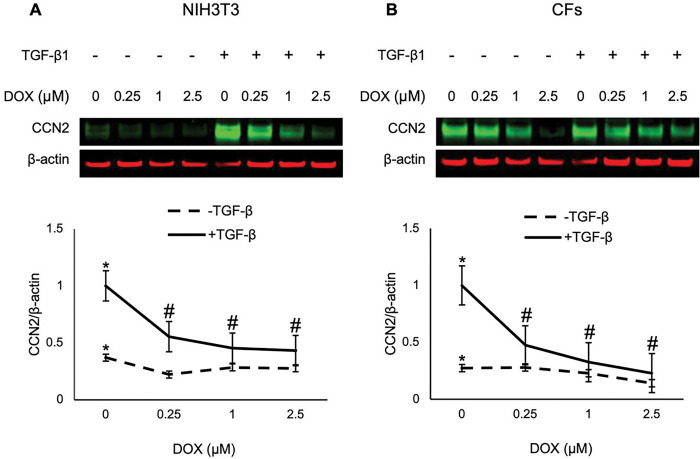
DOX inhibited TGF-β1 induced expression of CCN2 in a dose-dependent manner. The effect of DOX on CCN2 protein expression in NIH3T3 cells (A) and CFs (B) with and without 10 ng TGF-β1 stimulation was assessed. The cells were tested with increasing concentrations of DOX (0, 0,25, 1, or 2.5 μM), and 100 μg/mL ascorbic acid for 24 hours. Values represent mean ± standard error (n=4). Statistical analysis performed with two-way ANOVA. * *P*-value < 0.05 is compared between the control group and the treatment group stimulated with TGF-β1 without DOX, and # *P*-value < 0.05 are treatments compared to the treatment group stimulated with TGF-β1 without DOX.

In this study, we evaluated the expression of cellular communication network factor 2 (CCN2) protein in NIH3T3 and CFs treated with DOX (Fig. 2). A dose-dependent downregulation of CCN2 protein expression was observed in both cell lines treated for 24 hours with DOX and TGF-β1. These results suggest that DOX modulates the expression of CCN2 in fibroblasts and that DOX may play a role in ECM remodeling.

### DOX treatment inhibited the TGF-β-induced expression of BMP1 in NIH3T3 fibroblasts

**Figure 3:**
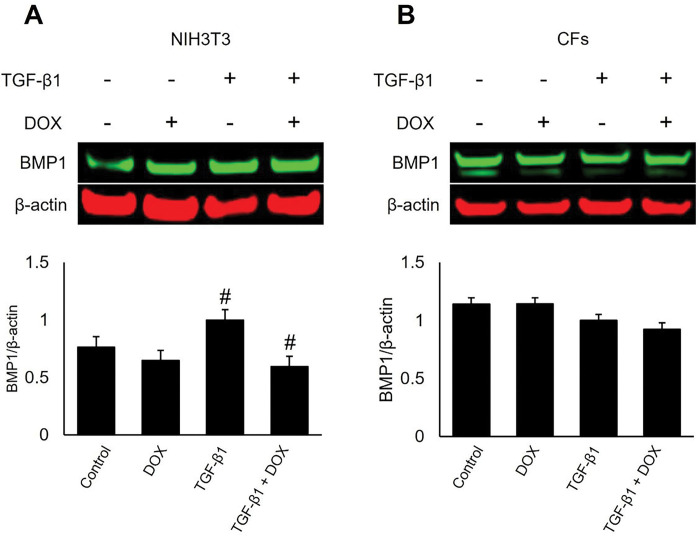
DOX inhibited the TGF-β1 induced expression of BMP-1 in NIH3T3 cells, while BMP-1 levels remained high in CFs. The effect of DOX on BMP1 protein expression in NIH3T3 cells (A) and cardiac fibroblasts (CFs) (B), with and without TGF-β1 stimulation was assessed. Cells were treated with 1 μM DOX and 100 μg/mL ascorbic acid for 24 hours, and protein extracts were collected for western blot analysis. Expression levels of BMP1 and the housekeeping protein β-actin were determined, and densitometry was performed for BMP1, normalized to β-actin. Error bars represent ± standard error (n=4). Statistical analysis was completed with Student’s t-test assuming unequal variance. # *P*-value < 0.05.

In this study, we examined the impact of DOX on the expression of BMP1 protein in NIH3T3 and CFs using western blot analysis. We observed significant changes in BMP1 protein levels in NIH3T3 cells induced by TGF-β1, but not in CFs (Fig. 3).

These findings indicate that DOX has a cell-specific effect on BMP1 protein levels in fibroblasts. Specifically, the observed changes in BMP1 levels in response to DOX treatment were evident in NIH3T3 cells. In CFs, BMP1 expression levels were higher under all conditions compared to NIH3T3 cells, indicating differences between the NIH3T3 cells and primary CFs. Further investigations are warranted to elucidate the underlying mechanisms and functional implications of these cell-specific effects on BMP1 expression in the context of TGF-β signaling and DOX treatments.

#### TGF-β1 induced the phosphorylation of SMAD2 after one hour in both NIH3T3 cells and CFs.

**Figure 4:**
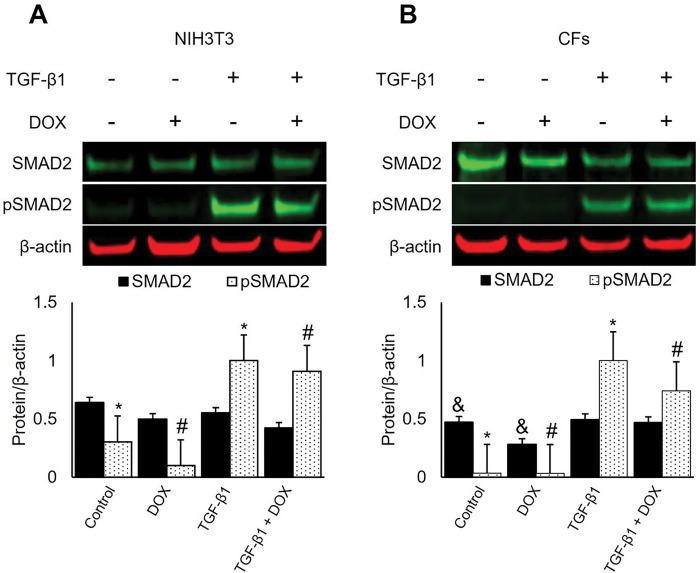
TGF-β1 induced the phosphorylation of SMAD2 in both NIH3T3 cells and CFs. DOX treatment diminished the extent of phosphorylation; however, the reduction did not reach statistical significance. The effect of DOX on SMAD2 and phosphorylated SMAD2 (pSMAD2) in NIH3T3 cells (a) and CFs (B) was assessed. Cells were treated with 1 mM DOX and 100 mg/mL ascorbic acid and stimulated with or without TGF-β1for one hour. Densitometry was performed on SMAD2, pSMAD2, and normalized to housekeeping protein β-actin. Values represent mean ± standard error (n=4). * *P*-value < 0.05 when compared to treatments without DOX and # *P*-value < 0.05 compared to treatments with DOX. & *P*-value < 0.05 are treatments with and without DOX without any TGF-β1 stimulation.

To investigate whether DOX affects the activation of the TGF-β/SMAD2 signaling pathway, we assessed SMAD2 and pSMAD2 in NIH3T3 and CFs treated with TGF-β1 and DOX for one hour ^51^. Our results indicated that TGF-β1 stimulation led to a significant increase in pSMAD2 in both cell lines, in agreement with previous findings ^52^. DOX treatment diminished the extent of phosphorylation of SMAD2, however the differences did not reach statistical significance during the one-hour treatment. Additionally, SMAD2 protein levels decreased slightly in response to DOX compared to control in CFs. This decrease was attenuated by exogenous TGF-β1 (Fig. 4).

### DOX treatment inhibited the TGF-β-induced COL1 protein expression in NIH3T3 fibroblasts and CFs

**Figure 5:**
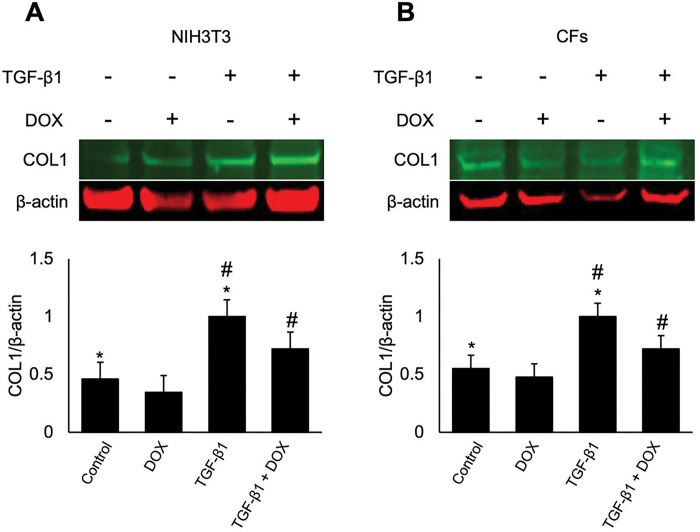
DOX inhibited the TGF-β1 induced expression of COL1 in NIH3T3 cells and CFs. Effect of DOX on COL1 protein expression in NIH3T3 cells (A) and CFs (B), with and without TGF-β1 stimulation was assessed. Cells were treated with 1 μM DOX and 100 μg/mL ascorbic acid for 24 hours, with or without 10 ng TGF-β1 stimulation, after which protein extracts were collected and subjected to western blotting to determine the expression levels of COL1 and the housekeeping protein β-actin. Densitometry was performed for COL1 and normalized to β-actin. Values represent mean ± standard error (n=4). *P*-values were determined using Student t-test assuming unequal variance * *P*-value < 0.05 control compared to treatment stimulated with TGF-β1 without DOX, and # *P*-value < 0.05 compared to both treatment groups treated with TGF-β1.

To evaluate the effects of DOX on COL1 protein expression in NIH3T3 and CFs, we conducted western blot analysis following a 24-hour treatment with DOX and TGF-β1. The findings demonstrated that TGF-β1 treatments led to an increase in COL1 protein. Additionally, DOX was able to inhibit the TGF-β1 induction of COL1 protein (Fig. 5). These findings indicate that DOX treatment hinders COL1 production, potentially affecting the deposition of mature collagen within the ECM surrounding cardiac fibroblasts.

The observed reduction in COL1 protein expression, resulting from DOX treatment indicated that further investigations are warranted to elucidate the underlying mechanisms through which DOX and TGF-β1 interact in CFs. Understanding these interactions could provide valuable insights into the development of strategies for mitigating the adverse effects of DOX on cardiac function and ECM production and remodeling.

#### DOX inhibited the TGF-β1-induced differential gene expression reduction of Atf4 and promoted differential increased gene expression of Id1, Runx1, Thbs1, Stat1, and Cdkn1a in NIH3T3 Fibroblasts

**Figure 6:**
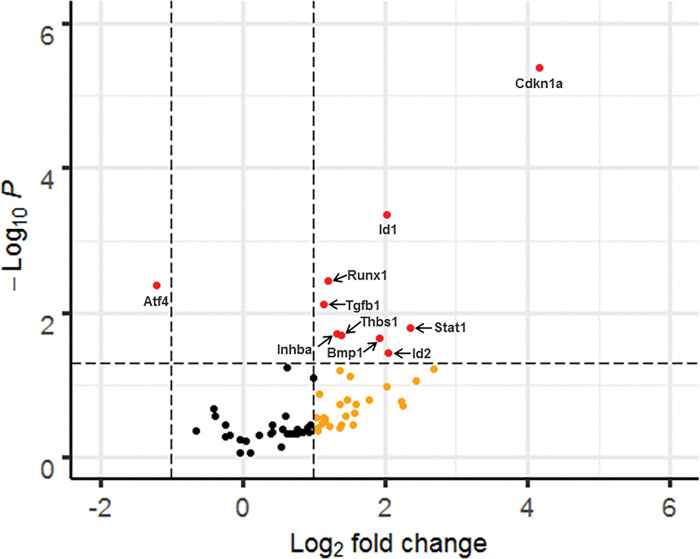
DOX inhibited the TGF-β1 induced expression of Atf4 and increased the expression of Cdkn1a, Id1, Id2, Runx1, Thbs1, Tgfb1, and Stat1 in NIH3T3 cells. Effects of DOX on the expression of selected genes in the TGFβ/BMP Signaling Pathways in NIH3T3 cells was assessed. Cells were treated with 10 ng TGF-β1 with or without 1 μM DOX for 24 hours.

In this study, we examined the effects of DOX on TGF-β1-induction of differential gene expression of negative and positive modulators of the TGF-β/BMP signaling pathway using qRT-PCR. DOX-induced differential expression of several gene in this pathway were observed in NIH3T3 cells, including upregulation of Id1, Id2, Runx1, Tgfb1, Inhba, Thbs1, Bmp1, and Stat1, and downregulation of Atf4 (Fig. 6, Table 1). In contrast, no significant gene alterations were observed in CFs under the same conditions (**Supplementary Fig. 1**). These findings indicate that DOX impacts TGF-β and BMP signaling in a cell type-specific manner.

## Discussion

This study provides data on the effects of DOX and TGF-β1 treatment on NIH3T3 and CFs, assessing specific target proteins of the TGF-β/BMP signaling pathway using western blot and qRT-PCR.

DOX is a widely used antibiotic with broad antitumor efficacy for treating hematopoietic and metastatic solid tumor cancers. However, its cardiotoxic side effect limits its clinical use. DOX-induced cardiotoxicity may manifest within hours of initial treatment and persist up to twenty years post-final administration, posing a potential lifelong risk for patients treated with DOX ^13–18^. Despite five decades of research, there is still no therapeutic option to reduce the risk of developing cardiotoxicity while maintaining its antitumor efficacy. Most research conducted on the matter has focused on cardiomyocytes, the chief cell type within the myocardium responsible for generating contractile force. However, the proper function of cardiomyocytes and the heart relies on other cell types, including CFs, which regulate the cardiac ECM through the TGF-β signaling pathway ^53^. The cardiac ECM provides scaffolding and support throughout the heart, facilitates force transmissions for proper pumping, and supplies neighboring cells with an extracellular environment. Therefore, CFs may be a potential therapeutic intervention target for reducing the risk of cardiotoxicity upon DOX treatment.

Cardiac fibroblasts are a crucial component of the myocardium and play a significant role in cardiac function. In CFs, the TGF-β1 signaling pathway regulates various cellular processes like differentiation, migration, proliferation, fibrosis, and ECM deposition. The TGF-β1 signaling pathway initiates with the binding of its ligand to the cell surface receptor complex, triggering the phosphorylation of SMAD2. Once phosphorylated, pSMAD2 forms a complex with SMAD4 and translocates into the nucleus, where it acts as a transcription factor to promote the expression of TGF-β target genes ^54–56^. In fibroblasts, SMAD2/pSMAD2 plays a crucial role in regulating the expression of various ECM genes, including collagens, CCN2, MMP2, and MMP9, which are involved in tissue remodeling processes following injury ^45,57,58^. Dysregulation of SMAD2/pSMAD2 has been implicated in several pathological conditions, particularly those related to cardiac fibrosis ^59–62^.

CCN2 is a multifunctional matricellular protein involved in many biological processes, including ECM production, migration, differentiation, angiogenesis, and apoptosis ^63–65^. CCN2 has been shown to be a downstream mediator of the TGF-β/SMAD signaling pathway, which play essential roles in the ECM synthesis and remodeling in the heart ^66,67^. Activation of these signaling pathways leads to the upregulation of CCN2 expression, which in turn stimulates the production of ECM components and inhibits ECM degradation, ultimately contributing to the maintenance of the cardiac structure and function ^68^.

BMP1 is a member of the astacin family of matrix metalloproteinases that play a critical role in the regulation of ECM synthesis and remodeling. In fibroblasts, BMP1 is involved in the regulation of TGF-β signaling by activating the TGF-β signaling pathway by cleaving the pro-domain of TGF-β precursors, which releases the active TGF-β ligand that then binds to its cell surface receptor complex and activates downstream SMAD proteins ^69,70^. BMP1 also cleaves several ECM proteins, including type I procollagen, which matures into the abundant COL1 ^71,72^.

COL1 represents a major component of the ECM in the heart, contributing to its structural integrity and tensile strength. Moreover, COL1 acts as a crucial scaffold for cardiac cells and actively participates in cardiac remodeling in response to injury or stress. Its presence is vital for the maintenance of cardiac function and the prevention of cardiovascular diseases ^73^. The expression of Col1 gene is regulated by the TGF-β signaling pathways, which stimulate the production of type I procollagen, subsequently undergoing maturation and deposition within the extracellular space ^74^.

The cardiac ECM plays a crucial role in maintaining cardiac homeostasis by continuously interacting with cellular elements, providing structural support, and coordinating cellular responses to ensure proper myocardial function. ECM regulation is achieved through the highly controlled synthesis of matrix proteins and proteolytic processing facilitated by MMPs and TIMPs. The dysregulation of the cardiac ECM can impair cardiac function and limit signaling between cells which can modulate cell behavior and lead to pathological changes in the heart.

This study focused on investigating the effect of DOX treatment on targets of the TGF-β signaling pathway in two mouse fibroblast cell lines. One of the key findings of this study is the dose-dependent downregulation of cellular CCN2 protein expression in both NIH3T3 fibroblasts and CFs upon DOX treatment. CCN2 plays a crucial role in ECM synthesis and remodeling in the heart. Activation of TGF-β signaling leads to the upregulation of CCN2 expression, which stimulates ECM production and inhibits ECM degradation. The background expression of CCN2 is usually very low, therefore, cells were stimulated with TGF-β1 (5 ng/mL) to induce CCN2 expression. The observed downregulation of CCN2 protein expression suggests that DOX treatment can modulate the expression of CCN2, potentially impacting ECM remodeling processes.

The TGF-β/SMAD signaling pathway is critical in regulating ECM gene expression and tissue remodeling. We evaluated SMAD2 protein expression and phosphorylation levels in response to DOX treatments. The treatment of TGF-β1 significantly increased the pSMAD2 expression in both NIH3T3 and CFs compared to treatment groups without the exogenous TGF-β1. A decrease in SMAD2 protein expression was observed in DOX-treated CFs compared to the control group. However, the response did not reach statistical significance in the TGF-β1 treated groups.

BMP1 is an enzyme involved in TGF-β signaling and ECM regulation. BMP1 is known to activate the TGF-β pathway and cleave ECM proteins such as type I procollagen. Our results showed a significant change in BMP1 protein levels in response to DOX treatment in NIH3T3 cells stimulated with TGF-β1. These findings suggest a cell-specific response to DOX treatment and indicate the need for further investigation to understand the underlying mechanisms and functional implications of these differential effects. Furthermore, while protein levels were decreased by DOX treatment, mRNA levels were slightly increased, indicating that transcription may be increased but translation is not.

COL1 protein is a major structural component of the cardiac ECM. COL1 provides structural integrity to the heart and plays a crucial role in cardiac remodeling. The TGF-β signaling pathway regulates COL1 expression, and our results demonstrated that DOX treatment, significantly reduced the TGF-β1-induced COL1 protein increase compared to TGF-β1 treatment alone. These findings suggest that DOX treatment impaired the production of collagen destined for the ECM, potentially affecting the cardiac remodeling processes and resulting in cardiac tissue integrity.

Moreover, this study examined the effects of DOX on gene transcription in the TGF-β and BMP signaling pathways. Significant alterations in gene expression for 10 genes were observed in NIH3T3 cells treated with DOX and TGF-β1, however, no differential gene expression was observed in CFs under the same conditions.

Specifically, we observed that Activating Transcription Factor 4 (Atf4), a gene that regulates various cellular processes, was significantly downregulated in NIH3T3 cells treated with TGF-β1 and DOX compared to TGF-β1 treatment alone. Prior reports have indicated a positive correlation between ATF4 protein levels and the expression of COL1, as evidenced by significant reductions in type 1 collagen biosynthesis in ATF4 knockout mice ^75^. These findings suggest a potential association between decreased expression of the Atf4 gene and reduced COL1 proteins expression. Further evidence is required to elucidate whether treatments of DOX affects ATF4 and the synthesis of ECM components in fibroblasts.

On the other hand, the inhibitor of DNA binding 1 (Id1), which is a transcriptional repressor that regulate genes involved in cellular processes such as proliferation, differentiation, and apoptosis, was significantly upregulated in the presence of DOX. Id1 is an inhibitor of the TGF-β-induced collagen expression in human dermal fibroblasts ^76^. Additionally, in stem-cell derived endothelial cells, TGF-β inhibition was found to be Id1 dependent ^77^. This would suggest that DOX negatively correlates with TGF-β signaling increasing Id1 expression and reducing collagen deposition.

We also found that Runt-related Transcription Factor 1 (Runx1) also known as AML1/ETO was upregulated. RUNX1 has been demonstrated in various cancer studies to inactivate the TGF-β1/SMAD signaling pathway ^78^. This pathway is responsible for regulating the expression of various ECM components such as collagens, fibronectins, MMPs and TIMPs.

Interestingly, there was an upregulation of Tgfb1, transforming growth factor beta 1, mRNA. Additionally, Thrombospondin 1 (Thbs1) mRNA was also found to be elevated in the presence of DOX + TGF-β1 compared to TGF-β1 treatment alone. Thbs1 promotes Tgfb1 expression in fibroblasts *in vitro*^79^. This suggests that DOX-induced Thbs1 mRNA expression explaining the increase observed in Tgfb1 mRNA expression. Further research is required to elucidate the effects of DOX on Tgfb1 and Thbs1 mRNA in fibroblast.

Signal Transducer and Activator of Transcription 1 (Stat1), a downstream target of the TGF-β signaling pathway, was upregulated in NIH3T3 cells upon treatment with DOX + TGF-β1 compared to TGF-β1 treatment alone. Stat1 has been observed to inhibit the myofibroblast phenotype in cells of fibroblastic origin *in vivo*^80^. This would suggest that DOX prevents the transformation of fibroblasts to myofibroblasts.

Lastly, cyclin dependent kinase inhibitor 1A (Cdkn1a) (also known as p21), is a senescence marker for oxidative stress that induces G2 arrest in the cell cycle in human fibroblasts ^81–84^.

The downregulation of Atf4 and upregulation of Id1, Runx1, and Stat1 in NIH3T3 cells suggest that DOX treatments may have an inhibitory effect on ECM deposition. These findings imply a potential reduction in the synthesis and deposition of ECM components. However, it is important to note that Cdkn1a was also upregulated in NIH3T3 cells. Cdkn1a is known to play a role in the transformation of fibroblasts into myofibroblasts. This finding appears contradictory to the previously mentioned Atf4, Id1, Runx1, and Stat1 in reducing ECM deposition as myofibroblasts are pro-fibrotic in nature and promote the biosynthesis and deposition of ECM. It is possible that the upregulation of Cdkn1a mRNA observed in this study was a result of oxidative stress caused by the 24-hour treatment of DOX, rather than a direct response to fibroblast transformation into myofibroblasts. Further investigation is required to fully elucidate the effects of DOX on these genes and clarify the underlying mechanisms.

In summary, this study provides valuable insights into the effects of DOX on fibroblast function, ECM remodeling, and the TGF-β signaling pathway involved in cardiac tissue homeostasis. The findings suggest that DOX treatment can modulate the expression of key proteins involved in TGF-β signaling, ECM synthesis, and remodeling, such as CCN2 and COL1. The differential effects observed in BMP1 protein levels highlight the cell-specific responses to DOX treatment. A small, but detectable increase in total SMAD2 protein was observed when comparing DOX treatment to DOX plus TGF-β1, and this may suggest that TGF-β1 as a co-treatment with DOX may lessen the side effects and may be a potential avenue for future investigation of therapeutic intervention. Further investigations are warranted to elucidate the underlying mechanisms and functional implications of these observations, which could contribute to the development of strategies for mitigating DOX-induced cardiotoxicity and preserving cardiac function in clinical settings.

## Materials and Methods

### Cell Culture

Cardiac fibroblasts (CFs; BALB/c mouse primary cardiac fibroblasts; Cell Biologics; BALB-5049) were cultured in fibroblast medium provided by Cell Biologics (M2267), which contained 0.5 mL fibroblasts growth factor (0.1% v/v), 0.5 mL hydrocortisone (0.1% v/v), 5 mL 100x antibiotic-antimycotic solution, 5 mL of 200 mM L-Glutamine, and 10% (v/v) fetal bovine serum. NIH3T3 cells (mouse embryonic fibroblasts; ATCC; CRL-1658) were cultured in high glucose Dulbecco’s Modified Eagle Medium (DMEM; Thermo Fisher Scientific; 11965092) supplemented with 10% (v/v) fetal bovine serum (Atlanta Biologicals; S22660), 5 mL of 10 units/L penicillin, and 5 mL 10 mg/mL streptomycin (Thermo Fisher Scientific; 15140122). Prior to seeding CFs, all culture vessels were pre-coated with a gelatin-based coating solution (Cell Biologics; 6950) for one hour. Both cell lines were maintained under standard conditions of 5% CO_2_ at 37°C and 95% humidity.

### Assessment of Cell Viability of Fibroblasts treated with DOX

Cell viability of fibroblast cell lines treated with DOX were assessed by alamarBlue assay (Thermo Fisher Scientific; A50100) at a seeding density of 1 × 10^4^ cells per well (100 μL) in 96-well flat bottom plates. The cells were treated with a series of DOX concentrations ranging from 0 to 50 μM for 24 hours. After 21 hours, 10 μL of 10X alamarBlue was added to each well, and incubated for three hours. Fluorescence conversion was measured using a Synergy H1 microplate reader (Biotek) with 560 nm excitation and 590 nm emission.

### Protein expression analysis by western blot

NIH3T3 and CFs were seeded at a density of 2 × 10^5^ cells per well (2 mL) in 6-well plates one day prior to their treatments. Treatments were administered for 24 hours, except for the SMAD2 phosphorylation study, which lasted for one hour. Cells were treated with DOX (1 μM; 0.25, 1, and 2.5 μM for CCN2 study) and 5 ng/mL TGF-β1 or the respective vehicle control, DMSO and 4 mM HCl, respectively. ECM synthesis was promoted with 100 μg/mL ascorbic acid during treatments ^50^. Cellular proteins were extracted using radioimmunoprecipitation assay (RIPA) buffer (Thermo Fisher Scientific; 89901) containing Halt^™^ protease and phosphatase inhibitors (Thermo Fisher Scientific; 78442). Protein concentration in the cell lysates were determined using a Pierce^™^ BCA protein assay kit (Thermo Fisher Scientific; 23225).

Protein samples (30 μg) were prepared with 4X protein sample loading buffer (LI-COR; 928-40004), and loaded onto 4–12% NuPAGE^™^ Bis-Tris gels (Thermo Fisher Scientific; NP0321BOX). The proteins were transferred onto pre-activated polyvinylidene difluoride (PVDF) membranes (Thermo Fisher Scientific; IB24001) using the iBlot^™^ 2 gel transfer device (Thermo Fisher Scientific; IB21002) with the following stepwise conditions: 1) 20 V, 1 mA, 1 minute, 2) 23 V, 1 mA, 4 minutes, and 3) 25 V, 1 mA, 2 minutes. The membranes were blocked in Intercept^®^ (tris-buffered saline; TBS) blocking buffer (LI-COR, 927-60003) for one hour and incubated in primary antibody (protein of interest and housekeeping primary antibody tandemly) prepared in Intercept^®^ T20 (TBS) antibody diluent (LI-COR, 927-65001) at 4°C overnight. After overnight incubation in the primary antibody, membranes were washed and incubated in a secondary antibody for one hour then washed again. Infrared detection of antibody binding on the membranes were scanned on the LI-COR Bioscience Odyssey CLx imaging system and analyzed for densitometry using LI-COR ImageStudio software.

### Primary and Secondary Antibodies

Primary antibodies used in this study were: recombinant rabbit anti-cellular communication network factor 2 (CCN2) monoclonal antibody diluted to a concentration of 1:1,000 (Abcam, ab209780); rabbit anti-bone morphogenetic protein 1 (BMP1) polyclonal antibody diluted to a concentration of 1:1,000 (Thermo Fisher Scientific; PA5-103660; RRID: AB_2852994); rabbit anti-collagen type I (COL1) polyclonal antibody diluted to a concentration of 1:1,000 (Thermo Fisher Scientific; PA1-26204; RRID: AB_2260734); rabbit anti-suppressors of mothers against decapentaplegic homolog 2 (SMAD2) polyclonal antibody diluted to a concentration of 1:1,000 (Thermo Fisher Scientific; 51-1300; RRID: AB_2533896); rabbit anti-phosphorylated SMAD2 (pSMAD2) monoclonal antibody diluted to a concentration of 1:1,000 (Thermo Fisher Scientific; MA5-15122; RRID: AB_10978317); and mouse anti-beta actin (β-actin) monoclonal antibody diluted to a concentration of 1:5,000 (Thermo Fisher Scientific; MA1-91399; RRID: AB_2273656).

For near-infrared fluorescence detection, IRDye^®^ 800CW donkey anti-rabbit and IRDye^®^ 680RD donkey anti-mouse secondary antibodies (LI-COR, 926-32213 and 926-68072, respectively) were used.

### Gene expression analysis by qRT-PCR

Both cell lines were seeded at a density of 2 × 10^5^ cells per well (2 mL) in 6 well plates. After one day, the cells were treated with 5 ng/mL TGF-β1, 100 μg/mL ascorbic acid, 1μM DOX or their vehicle controls for 24 hours. Total RNA was extracted from the cell lines using the RNeasy Mini Kit (Qiagen; 217004). RNA concentrations were measured using a NanoDrop 2000 spectrophotometer (Thermo Fisher Scientific; ND2000USCAN). Subsequently, the RT^2^ first strand kit (Qiagen; 330401) was utilized to perform reverse transcription of 100 ng RNA from each sample into cDNA, in accordance with the manufacturer’s instructions. The cDNA was mixed with SYBR Green mastermix (Qiagen; 330503) for fluorescence detection using a thermocycler (LightCycler 96, Roche). We employed a TGF-β/BMP signaling pathway RT^2^ Profiler^™^ PCR 96 well plate containing primers specific for genes related to the TGF-β/BMP signaling cascade, RNA controls, and housekeeping genes (Qiagen; PAMM-035ZF-12; 330231). The 96-well plate was loaded with 25 μL of cDNA per well. The following thermal profile was applied: 1 cycle at 95°C for 10 minutes and 45 cycles at 95°C for 15 seconds, then 60°C for one minute. A web-based tool from Qiagen, RT^2^ Profiler PCR Data Analysis, was used for differential gene expression analysis.

### Statistical Analysis

The values were presented as mean ± SE. Differences between the two groups were evaluated using the Student t-test. Differences among multiple groups were evaluated using a two-way analysis of variance (ANOVA) with Dunnett’s test. The level of significance was selected to be P < 0.05. R statistics software was used to perform the statistical analysis.

## Figures and Tables

**Table 1:** Up/Downregulated Genes in NIH3T3 Cells Treated with DOX and TGF-β1

Gene Symbol	Gene Name	Alias	Regulation
Atf4	Activating Transcription Factor 4	Creb-2. Taxreb67, Txreb	Down
Bmp1	Bone Morphogenetic Protein 1	Bmp-1, Pcolc, Tolloid-like	Up
Cdkn1a	Cyclin Dependent Kinase Inhibitor 1A	p21, Cap20, Cip1, Waf1, Sd1, p21cip1, cdkn1	Up
Id1	Inhibitor of DNA Binding 1	Bhlhb24	Up
Id2	Inhibitor of DNA Binding 2	Bhlhb26, Gig8	Up
Inhba	Inhibin Subunit Beta A	Edf, Activin beta-A chain	Up
Runx1	RUNX Family Transcription Factor 1	Amlcr1, Cbfa2, Aml1, Aml1/ETO, Pebp2a2	Up
Stat1	Signal Transducer and Activator of Transcription 1	Stat91, Isgf-3	Up
Tgfb1	Transforming Groth Factor Beta 1	Tgfbeta, Tgfb, Ced, Dpd1	Up
Thbs1	Thrombospondin 1	Tsp1, Tsp, Thbs-1, Tsp-1	Up

## Data Availability

The authors confirm that the data supporting the findings of this study are available within the article and its supplementary materials.

## References

[1] Zhang S. Identification of the molecular basis of doxorubicin-induced cardiotoxicity. Nat Med 18, 1639–1642 (2012). 23104132 10.1038/nm.2919

[2] Weaver K. E. Cardiovascular risk factors among long-term survivors of breast, prostate, colorectal, and gynecologic cancers: a gap in survivorship care? J Cancer Surviv 7, 253–261 (2013). 23417882 10.1007/s11764-013-0267-9 PMC3756807

[3] Aleman B. M. P. Cardiovascular disease after cancer therapy. EJC Suppl 12, 18–28 (2014). 26217163 10.1016/j.ejcsup.2014.03.002 PMC4250533

[4] Giza D. E., Iliescu G., Hassan S., Marmagkiolis K. & Iliescu C. Cancer as a Risk Factor for Cardiovascular Disease. Curr Oncol Rep 19, (2017). 10.1007/s11912-017-0601-x 28421481

[5] Doxorubicin Hydrochloride - NCI. https://www.cancer.gov/about-cancer/treatment/drugs/doxorubicinhydrochloride.

[6] Sampaio D. P. S., Silva J. B. M., do Carmo Rassi D., Freitas A. F. & Rassi S. Echocardiographic strategy for early detection of cardiotoxicity of doxorubicin: a prospective observational study. Cardio-Oncology 8, 1–9 (2022). 36183108 10.1186/s40959-022-00143-0 PMC9526268

[7] Dobson R. BSE and BCOS Guideline for Transthoracic Echocardiographic Assessment of Adult Cancer Patients Receiving Anthracyclines and/or Trastuzumab. JACC CardioOncol 3, 1–16 (2021). 34396303 10.1016/j.jaccao.2021.01.011 PMC8352267

[8] Tak T., Jaekel C. M., Gharacholou S. M., Dworak M. W. & Marshall S. A. Measurement of Ejection Fraction by Cardiac Magnetic Resonance Imaging and Echocardiography to Monitor Doxorubicin-Induced Cardiotoxicity. International Journal of Angiology 29, 45–51 (2020). 10.1055/s-0039-1697921 PMC7054057 32132816

[9] Zamorano J. L. 2016 ESC Position Paper on cancer treatments and cardiovascular toxicity developed under the auspices of the ESC Committee for Practice Guidelines: The Task Force for cancer treatments and cardiovascular toxicity of the European Society of Cardiology (ESC). Eur J Heart Fail 19, 9–42 (2017). 27565769 10.1002/ejhf.654

[10] Plana J. C. Expert consensus for multimodality imaging evaluation of adult patients during and after cancer therapy: a report from the American Society of Echocardiography and the European Association of Cardiovascular Imaging. J Am Soc Echocardiogr 27, 911–939 (2014). 25172399 10.1016/j.echo.2014.07.012

[11] Thavendiranathan P. Reproducibility of echocardiographic techniques for sequential assessment of left ventricular ejection fraction and volumes: Application to patients undergoing cancer chemotherapy. J Am Coll Cardiol 61, 77–84 (2013). 23199515 10.1016/j.jacc.2012.09.035

[12] Martín M. Minimizing Cardiotoxicity While Optimizing Treatment Efficacy with Trastuzumab: Review and Expert Recommendations. Oncologist 14, 1–11 (2009). 10.1634/theoncologist.2008-0137 19147689

[13] Steinherz L. J., Steinherz P. G., Tan C. T. C., Heller G. & Murphy M. L. Cardiac Toxicity 4 to 20 Years After Completing Anthracycline Therapy. JAMA 266, 1672–1677 (1991). 1886191

[14] Curigliano G. Cardiotoxicity of anticancer treatments: Epidemiology, detection, and management. CA Cancer J Clin 66, 309–325 (2016). 26919165 10.3322/caac.21341

[15] Howlader N. Improved estimates of cancer-specific survival rates from population-based data. J Natl Cancer Inst 102, 1584–1598 (2010). 20937991 10.1093/jnci/djq366 PMC2957430

[16] Wouters K. A., Kremer L. C. M., Miller T. L., Herman E. H. & Lipshultz S. E. Protecting against anthracycline-induced myocardial damage: a review of the most promising strategies. Br J Haematol 131, 561–578 (2005). 16351632 10.1111/j.1365-2141.2005.05759.x

[17] Martin M. Adjuvant docetaxel for node-positive breast cancer. N Engl J Med 352, 2302–2313 (2005). 15930421 10.1056/NEJMoa043681

[18] Barrett-Lee P. J. Expert opinion on the use of anthracyclines in patients with advanced breast cancer at cardiac risk. Ann Oncol 20, 816–827 (2009). 19153118 10.1093/annonc/mdn728

[19] Swain S. M., Whaley F. S. & Ewer M. S. Congestive heart failure in patients treated with doxorubicin: a retrospective analysis of three trials. Cancer 97, 2869–2879 (2003). 12767102 10.1002/cncr.11407

[20] Goto S. Doxorubicin-induced DNA intercalation and scavenging by nuclear glutathione S-transferase pi. FASEB J 15, 2702–2714 (2001). 11726546 10.1096/fj.01-0376com

[21] Yao F. Nanopore single-molecule analysis of DNA-doxorubicin interactions. Anal Chem 87, 338–342 (2015). 25493921 10.1021/ac503926g

[22] Agudelo D., Bourassa P., Bérubé G. & Tajmir-Riahi H. A. Intercalation of antitumor drug doxorubicin and its analogue by DNA duplex: structural features and biological implications. Int J Biol Macromol 66, 144–150 (2014). 24560949 10.1016/j.ijbiomac.2014.02.028

[23] Mobaraki M. Molecular Mechanisms of Cardiotoxicity: A Review on Major Side-effect of Doxorubicin. Indian J Pharm Sci 79, 335–344 (2017).

[24] Ramachandran C., Samy T. S. A., Xiao Ling Huang, Zhao Kang Yuan & Krishan, A. Doxorubicin-induced DNA breaks, topoisomerase II activity and gene expression in human melanoma cells. Biochem Pharmacol 45, 1367–1371 (1993). 8385463 10.1016/0006-2952(93)90293-6

[25] Bodley A. DNA Topoisomerase II-mediated Interaction of Doxorubicin and Daunorubicin Congeners with DNA1. Cancer Res 5969–5978 (1989). 2551497

[26] Davies K. J. A., Doroshow J. H. & Hochstein P. Mitochondrial NADH dehydrogenase-catalyzed oxygen radical production by adriamycin, and the relative inactivity of 5-iminodaunorubicin. FEBS Lett 153, 227–230 (1983). 6298008 10.1016/0014-5793(83)80153-7

[27] Mukhopadhyay P., Rajesh M., Yoshihiro K., Haskó G. & Pacher P. Simple quantitative detection of mitochondrial superoxide production in live cells. Biochem Biophys Res Commun 358, 203 (2007). 17475217 10.1016/j.bbrc.2007.04.106 PMC2228267

[28] Wu J. Calcium Overload or Underload? The Effects of Doxorubicin on the Calcium Dynamics in Guinea Pig Hearts. Biomedicines 10, (2022). 10.3390/biomedicines10092197 PMC9496179 36140298

[29] Ondrias K., Borgatta L., Kim D. H. & Ehrlich B. E. Biphasic effects of doxorubicin on the calcium release channel from sarcoplasmic reticulum of cardiac muscle. Circ Res 67, 1167–1174 (1990). 2171802 10.1161/01.res.67.5.1167

[30] Šimůnek T. Anthracycline-induced cardiotoxicity: overview of studies examining the roles of oxidative stress and free cellular iron. Pharmacol Rep 61, 154–171 (2009). 19307704 10.1016/s1734-1140(09)70018-0

[31] Rao V. A. Iron Chelators with Topoisomerase-Inhibitory Activity and Their Anticancer Applications. Antioxid Redox Signal 18, 930 (2013). 22900902 10.1089/ars.2012.4877 PMC3557438

[32] Wei S. Involvement of ROS/NLRP3 Inflammasome Signaling Pathway in Doxorubicin-Induced Cardiotoxicity. Cardiovasc Toxicol 20, 507–519 (2020). 32607760 10.1007/s12012-020-09576-4

[33] Shi S., Chen Y., Luo Z., Nie G. & Dai Y. Role of oxidative stress and inflammation-related signaling pathways in doxorubicin-induced cardiomyopathy. Cell Communication and Signaling 2023 21:1 21, 1–20 (2023). 36918950 10.1186/s12964-023-01077-5 PMC10012797

[34] Reuter S., Gupta S. C., Chaturvedi M. M. & Aggarwal B. B. Oxidative stress, inflammation, and cancer: How are they linked? Free Radic Biol Med 49, 1603–1616 (2010). 20840865 10.1016/j.freeradbiomed.2010.09.006 PMC2990475

[35] Fang J., Seki T. & Maeda H. Therapeutic strategies by modulating oxygen stress in cancer and inflammation. Adv Drug Deliv Rev 61, 290–302 (2009). 19249331 10.1016/j.addr.2009.02.005

[36] Sangweni N. F. Prevention of Anthracycline-Induced Cardiotoxicity: The Good and Bad of Current and Alternative Therapies. Front Cardiovasc Med 9, (2022). 10.3389/fcvm.2022.907266 PMC9257015 35811736

[37] Zhou P. & Pu W. T. Recounting Cardiac Cellular Composition. Circ Res 118, 368–370 (2016). 26846633 10.1161/CIRCRESAHA.116.308139 PMC4755297

[38] Tyaci S. C. Extracellular Matrix Regulation of Metalloproteinase and Antiproteinase in Human Heart Fibroblast Cells. J Cell Physiol 167, 137–147 (1996). 8698831 10.1002/(SICI)1097-4652(199604)167:1<137::AID-JCP16>3.0.CO;2-8

[39] Turner N. A. & Porter K. E. Regulation of myocardial matrix metalloproteinase expression and activity by cardiac fibroblasts. IUBMB Life 64, 143–150 (2012). 22215527 10.1002/iub.594

[40] Philips N., Bashey R. I. & Jimenez S. A. Collagen and fibronectin expression in cardiac fibroblasts from hypertensive rats. Cardiovasc Res 28, 1342–1347 (1994). 7954643 10.1093/cvr/28.9.1342

[41] Eghbali M. & Weber K. T. Collagen and the myocardium: fibrillar structure, biosynthesis and degradation in relation to hypertrophy and its regression. Mol Cell Biochem 96, 1–14 (1990). 2146489 10.1007/BF00228448

[42] Bagchi R. A., Lin J., Wang R. & Czubryt M. P. Regulation of fibronectin gene expression in cardiac fibroblasts by scleraxis. Cell Tissue Res 366, 381–391 (2016). 27324126 10.1007/s00441-016-2439-1

[43] Frangogiannis N. G. Transforming growth factor–β in tissue fibrosis. Journal of Experimental Medicine vol. 217 Preprint at 10.1084/jem_20190103 (2020). PMC7062524 32997468

[44] Saadat S. Pivotal Role of TGF-β/Smad Signaling in Cardiac Fibrosis: Non-coding RNAs as Effectual Players. Frontiers in Cardiovascular Medicine vol. 7 Preprint at 10.3389/fcvm.2020.588347 (2021). PMC7868343 33569393

[45] Leask A. TGFβ, cardiac fibroblasts, and the fibrotic response. Cardiovasc Res 74, 207–212 (2007). 16919613 10.1016/j.cardiores.2006.07.012

[46] Travers J. G., Kamal F. A., Robbins J., Yutzey K. E. & Blaxall B. C. Cardiac Fibrosis. Circ Res 118, 1021–1040 (2016). 26987915 10.1161/CIRCRESAHA.115.306565 PMC4800485

[47] Baudino T. A., Carver W., Giles W. & Borg T. K. Cardiac fibroblasts: Friend or foe? Am J Physiol Heart Circ Physiol 291, 1015–1026 (2006). 10.1152/ajpheart.00023.2006 16617141

[48] Cleutjens J. P. M., Verluyten M. J. A., Smits J. F. M. & Daemen M. J. A. P. Collagen remodeling after myocardial infarction in the rat heart. Am J Pathol 147, 325 (1995). 7639329 PMC1869816

[49] Cohn J. N., Ferrari R. & Sharpe N. Cardiac remodeling—concepts and clinical implications: a consensus paper from an international forum on cardiac remodeling. J Am Coll Cardiol 35, 569–582 (2000). 10716457 10.1016/s0735-1097(99)00630-0

[50] Marinkovic M. Optimization of extracellular matrix production from human induced pluripotent stem cell-derived fibroblasts for scaffold fabrication for application in wound healing. J Biomed Mater Res A 109, 1803–1811 (2021). 33755305 10.1002/jbm.a.37173

[51] Pierreux C. E., Nicolás F. J. & Hill C. S. Transforming Growth Factor β-Independent Shuttling of Smad4 between the Cytoplasm and Nucleus. Mol Cell Biol 20, 9041–9054 (2000). 11074002 10.1128/mcb.20.23.9041-9054.2000 PMC86557

[52] Albers R. E., Selesniemi K., Natale D. R. C. & Brown T. L. TGF-β induces Smad2 Phosphorylation, ARE Induction, and Trophoblast Differentiation. Int J Stem Cells 11, 111 (2018). 29699384 10.15283/ijsc17069 PMC5984065

[53] Porter K. E. & Turner N. A. Cardiac fibroblasts: At the heart of myocardial remodeling. Pharmacol Ther 123, 255–278 (2009). 19460403 10.1016/j.pharmthera.2009.05.002

[54] Hata A. & Chen Y. G. TGF-β Signaling from Receptors to Smads. Cold Spring Harb Perspect Biol 8, (2016). 10.1101/cshperspect.a022061 PMC5008074 27449815

[55] Moustakas A. & Heldin C. H. The regulation of TGFβ signal transduction. Development 136, 3699–3714 (2009). 19855013 10.1242/dev.030338

[56] Massagué J. TGF-β SIGNAL TRANSDUCTION. Annu. Rev. Biochem 67, 753–91 (1998). 9759503 10.1146/annurev.biochem.67.1.753

[57] Huang S. The role of Smad2 and Smad3 in regulating homeostatic functions of fibroblasts in vitro and in adult mice. Biochim Biophys Acta Mol Cell Res 1867, (2020). 10.1016/j.bbamcr.2020.118703 PMC7261645 32179057

[58] Phanish M. K., Wahab N. A., Colville-Nash P., Hendry B. M. & Dockrell M. E. C. The differential role of Smad2 and Smad3 in the regulation of pro-fibrotic TGFβ1 responses in human proximal-tubule epithelial cells. Biochemical Journal 393, 601 (2006). 16253118 10.1042/BJ20051106 PMC1360711

[59] Khalil H. Fibroblast-specific TGF-β–Smad2/3 signaling underlies cardiac fibrosis. J Clin Invest 127, 3770–3783 (2017). 28891814 10.1172/JCI94753 PMC5617658

[60] Hanna A., Humeres C. & Frangogiannis N. G. The role of Smad signaling cascades in cardiac fibrosis. Cell Signal 77, 109826 (2021). 33160018 10.1016/j.cellsig.2020.109826 PMC7727442

[61] Hao J. Elevation of expression of Smads 2, 3, and 4, decorin and TGF-beta in the chronic phase of myocardial infarct scar healing. J Mol Cell Cardiol 31, 667–678 (1999). 10198196 10.1006/jmcc.1998.0902

[62] Huang S. Distinct roles of myofibroblast-specific Smad2 and Smad3 signaling in repair and remodeling of the infarcted heart. J Mol Cell Cardiol 132, 84–97 (2019). 31085202 10.1016/j.yjmcc.2019.05.006 PMC6579679

[63] Brigstock D. R. The CCN family: a new stimulus package. Journal of Endocrinology vol. 178 http://www.endocrinology.org (2003). 10.1677/joe.0.1780169 12904165

[64] Pi L. Connective tissue growth factor with a novel fibronectin binding site promotes cell adhesion and migration during rat oval cell activation. Hepatology 47, 996–1004 (2008). 18167060 10.1002/hep.22079 PMC3130595

[65] Chen Z. Connective Tissue Growth Factor: From Molecular Understandings to Drug Discovery. Front Cell Dev Biol 8, 1239 (2020). 10.3389/fcell.2020.593269 PMC7658337 33195264

[66] Dobaczewski M., Chen W. & Frangogiannis N. G. Transforming Growth Factor (TGF)-β signaling in cardiac remodeling. J Mol Cell Cardiol 51, 600 (2011). 21059352 10.1016/j.yjmcc.2010.10.033 PMC3072437

[67] Grotendorst G. R. Connective tissue growth factor: A mediator of TGf-β action on fibroblasts. Cytokine Growth Factor Rev 8, 171–179 (1997). 9462483 10.1016/s1359-6101(97)00010-5

[68] Dorn L. E., Petrosino J. M., Wright P. & Accornero F. CTGF/CCN2 is an autocrine regulator of cardiac fibrosis. J Mol Cell Cardiol 121, 205 (2018). 30040954 10.1016/j.yjmcc.2018.07.130 PMC6260782

[69] Massagué J., Seoane J. & Wotton D. Smad transcription factors. Genes Dev 19, 2783–2810 (2005). 16322555 10.1101/gad.1350705

[70] Ge G. & Greenspan D. S. BMP1 controls TGFβ1 activation via cleavage of latent TGFβ-binding protein. J Cell Biol 175, 111 (2006). 17015622 10.1083/jcb.200606058 PMC2064503

[71] Kessler E., Takahara K., Biniaminov L., Brusel M. & Greenspan D. S. Bone morphogenetic protein-1: the type I procollagen C-proteinase. Science 271, 360–362 (1996). 8553073 10.1126/science.271.5247.360

[72] N’Diaye E. N. Extracellular BMP1 is the major proteinase for COOH-terminal proteolysis of type I procollagen in lung fibroblasts. Am J Physiol Cell Physiol 320, C162–C174 (2021). 33206546 10.1152/ajpcell.00012.2020

[73] Cowling R. T., Kupsky D., Kahn A. M., Daniels L. B. & Greenberg B. H. Mechanisms of cardiac collagen deposition in experimental models and human disease. Transl Res 209, 138 (2019). 30986384 10.1016/j.trsl.2019.03.004 PMC6996650

[74] Petrov V. V., Fagard R. H. & Lijnen P. J. Stimulation of collagen production by transforming growth factor-beta1 during differentiation of cardiac fibroblasts to myofibroblasts. Hypertension 39, 258–263 (2002). 11847194 10.1161/hy0202.103268

[75] Verginadis I. I. A stromal Integrated Stress Response activates perivascular cancer-associated fibroblasts to drive angiogenesis and tumour progression. Nature Cell Biology 2022 24:6 24, 940–953 (2022). 35654839 10.1038/s41556-022-00918-8 PMC9203279

[76] Je Y. J. Inhibitory role of Id1 on TGF-β-induced collagen expression in human dermal fibroblasts. Biochem Biophys Res Commun 444, 81–85 (2014). 24434151 10.1016/j.bbrc.2014.01.010

[77] James D. Expansion and maintenance of human embryonic stem cell–derived endothelial cells by TGFβ inhibition is Id1 dependent. Nature Biotechnology 2010 28:2 28, 161–166 (2010). 10.1038/nbt.1605 PMC2931334 20081865

[78] Jakubowiak A. Inhibition of the transforming growth factor β1 signaling pathway by the AML1/ETO leukemia-associated fusion protein. Journal of Biological Chemistry 275, 40282–40287 (2000). 11032826 10.1074/jbc.C000485200

[79] Jiang D., Guo B., Lin F., Hui Q. & Tao K. Effect of THBS1 on the Biological Function of Hypertrophic Scar Fibroblasts. Biomed Res Int 2020, (2020). 10.1155/2020/8605407 PMC7744174 33376743

[80] Medley S. C., Rathnakar B. H., Georgescu C., Wren J. D. & Olson L. E. Fibroblast-specific Stat1 deletion enhances the myofibroblast phenotype during tissue repair. Wound Repair and Regen 28, 448–459 (2020). 10.1111/wrr.12807 PMC7321860 32175700

[81] Muto J. Highly concentrated trehalose induces prohealing senescence-like state in fibroblasts via CDKN1A/p21. Communications Biology 2023 6:1 6, 1–18 (2023). 36609486 10.1038/s42003-022-04408-3 PMC9822918

[82] Roninson I. B. Oncogenic functions of tumour suppressor p21Waf1/Cip1/Sdi1: association with cell senescence and tumour-promoting activities of stromal fibroblasts. Cancer Lett 179, 1–14 (2002). 11880176 10.1016/s0304-3835(01)00847-3

[83] Martins S. G., Zilhão R., Thorsteinsdóttir S. & Carlos A. R. Linking Oxidative Stress and DNA Damage to Changes in the Expression of Extracellular Matrix Components. Front Genet 12, 673002 (2021). 34394183 10.3389/fgene.2021.673002 PMC8358603

[84] Karimian A., Ahmadi Y. & Yousefi B. Multiple functions of p21 in cell cycle, apoptosis and transcriptional regulation after DNA damage. DNA Repair (Amst) 42, 63–71 (2016). 27156098 10.1016/j.dnarep.2016.04.008

